# Identification of TbPBN1 in *Trypanosoma brucei* reveals a conserved heterodimeric architecture for glycosylphosphatidylinositol‐mannosyltransferase‐I

**DOI:** 10.1111/mmi.14859

**Published:** 2021-12-25

**Authors:** Andrew Cowton, Peter Bütikofer, Robert Häner, Anant K. Menon

**Affiliations:** ^1^ Institute of Biochemistry and Molecular Medicine University of Bern Bern Switzerland; ^2^ Department of Chemistry, Biochemistry and Pharmaceutical Sciences University of Bern Bern Switzerland; ^3^ Department of Biochemistry Weill Cornell Medical College New York New York USA

**Keywords:** Endoplasmic reticulum (ER), glycosylphosphatidylinositol (GPI), glycosyltransferase, mannosyltransferase, *Trypanosoma brucei*

## Abstract

Glycosylphosphatidylinositol (GPI)‐anchored proteins are found in all eukaryotes and are especially abundant on the surface of protozoan parasites such as *Trypanosoma brucei*. GPI‐mannosyltransferase‐I (GPI‐MT‐I) catalyzes the addition of the first of three mannoses that make up the glycan core of GPI. Mammalian and yeast GPI‐MT‐I consist of two essential subunits, the catalytic subunit PIG‐M/Gpi14 and the accessory subunit PIG‐X/Pbn1(mammals/yeast). *T. brucei* GPI‐MT‐I has been highlighted as a potential antitrypanosome drug target but has not been fully characterized. Here, we show that *T. brucei* GPI‐MT‐I also has two subunits, TbGPI14 and TbPBN1. Using TbGPI14 deletion, and TbPBN1 RNAi‐mediated depletion, we show that both proteins are essential for the mannosyltransferase activity needed for GPI synthesis and surface expression of GPI‐anchored proteins. In addition, using native PAGE and co‐immunoprecipitation analyses, we demonstrate that TbGPI14 and TbPBN1 interact to form a higher‐order complex. Finally, we show that yeast Gpi14 does not restore GPI‐MT‐I function in TbGPI14 knockout trypanosomes, consistent with previously demonstrated species specificity within GPI‐MT‐I subunit associations. The identification of an essential trypanosome GPI‐MT‐I subcomponent indicates wide conservation of the heterodimeric architecture unusual for a glycosyltransferase, leaving open the question of the role of the noncatalytic TbPBN1 subunit in GPI‐MT‐I function.

## Graphical abstract

GPI‐mannosyltransferase‐I (GPI‐MT‐I) catalyzes the addition of the first of three mannoses during glycosylphosphatidylinositol (GPI) synthesis. Mammalian and yeast GPI‐MT‐I are heterodimers of the catalytic PIG‐M/Gpi14 and accessory PIG‐X/Pbn1 subunits. Here we show that GPI‐MT‐I from *Trypanosoma brucei* also consists of two subunits, TbGPI14 and TbPBN1, and both proteins are required for GPI‐MT‐I activity and GPI synthesis. This is the first report of a protozoan PIG‐X/Pbn1 protein and suggests wide conservation of a heterodimeric GPI‐MT‐I.

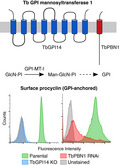

**Abbreviations**
Cas9: CRISPR‐associated protein 9
CRISPR: clustered regularly interspaced short palindromic repeats
ER: endoplasmic reticulum
GlcN: glucosamine
GlcNAc: N‐acetylglucosamine
GPI: glycosylphosphatidylinositol
GPI‐MT‐I: GPI‐mannosyltransferase‐I
PI: phosphatidylinositol

## 1 INTRODUCTION

Glycosylphosphatidylinositol (GPI)‐anchored proteins are ubiquitous in eukaryotes, from budding yeast and humans to the early diverging protist *Trypanosoma brucei*, the causative agent of African sleeping sickness (Kinoshita, 2020; Orlean & Menon, 2007). The GPI anchor, comprising the conserved core structure ethanolamine‐PO_4_‐(6)‐Manα–(1‐2)‐Manα–(1‐6)‐Manα‐(1‐4)‐GlcNα‐(1‐4)–*myo*‐inositol‐phospholipid, is synthesized in the endoplasmic reticulum (ER) by the sequential addition of components to phosphatidylinositol (PI) (Masterson et al., 1989; Menon et al., 1990b). In all eukaryotes, GPI synthesis begins on the cytoplasmic side of the ER with the transfer of GlcNAc from UDP‐GlcNAc to PI (Vidugiriene & Menon, 1993). GlcNAc‐PI is then de‐*N*‐acetylated (Doering et al., 1989; Menon et al., 1990b) and the resulting GlcN‐PI is flipped across the membrane (Vishwakarma & Menon, 2005). In yeast and mammals, GlcN‐PI is acylated on the inositol residue before the sequential addition of mannose (from dolichyl‐phosphate‐mannose) (Menon et al., 1990a) and ethanolamine phosphate (from phosphatidylethanolamine) (Menon et al., 1993; Menon & Stevens, 1992), whereas in the protozoan parasite *T. brucei*, the first mannose is transferred to GlcN‐PI prior to inositol acylation (Güther & Ferguson, 1995). The first mannosyl transfer is catalyzed by GPI‐mannosyltransferase‐I (GPI‐MT‐I), and the different substrate specificities of trypanosome and mammalian GPI‐MT‐I (GlcN‐PI vs. GlcN‐(acyl)PI, respectively) has led to the design of trypanosome‐specific GPI‐MT‐I inhibitors (Smith et al., 1999), making GPI‐MT‐I an attractive target for antitrypanosome drug discovery.

Yeast and mammalian GPI‐MT‐I consist of two essential subunits, the catalytic subunit Gpi14/PIG‐M and accessory subunit Pbn1/PIG‐X (Ashida et al., 2005). Both subunits are essential for GPI‐MT‐I activity in mammals and yeast, and the association between the subunits is species specific, that is, yeast Gpi14 cannot function with human PIG‐X and vice versa (Ashida et al., 2005; Kim et al., 2007). *T. brucei* GPI14 was previously cloned based on similarity to mammalian PIG‐M (Maeda et al., 2001), and *T. brucei* GPI‐MT‐I function has been studied in cell‐free systems (Smith et al., 1997, 1999). However, to date, no *T. brucei* Pbn1/PIG‐X homolog has been identified, and it was previously reported that this GPI‐MT‐I subunit may be absent from the *T. brucei* genome (Cardoso et al., 2013; Hong & Kinoshita, 2009; Kim et al., 2007). This absence either suggests that *T. brucei* GPI‐MT‐I has no accessory subunit, or that this subunit is too diverged to be detected by similarity searches, either explanation a possible reflection of the different substrate specificities of *T. brucei* and yeast/mammal GPI‐MT‐I. However, the observation that *T. brucei* GPI14 cannot rescue the lethality of Gpi14‐deficient yeast, and cannot display mannosyltransferase activity in Gpi14‐deficient yeast microsomes (Kim et al., 2007), suggests that TbGPI14 may also require a distinct second subunit for function. Here we address this point by demonstrating experimentally that TbGPI14 is responsible for GPI‐MT‐I activity, and therefore is essential for GPI synthesis, in *T. brucei*. We further identify and characterize a TbPBN1 homolog, which we show to be an essential subunit of GPI‐MT‐I in *T. brucei*.

## 2 RESULTS

### 2.1 TbGPI14 is dispensable for normal growth of procyclic form trypanosomes

**FIGURE 1:**
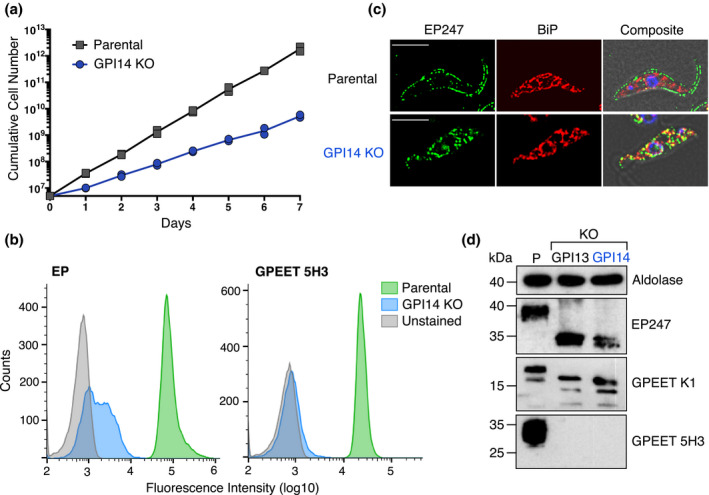
Loss of GPI‐anchored procyclins from TbGPI14 null cells. (a) Growth of parental and TbGPI14 KO cells counted every 24 hr for 7 days. Both cell lines were counted in two independent experiments and all data points are plotted. (b) Flow cytometry analysis of surface EP and GPEET expression on parental (green) and TbGPI14 KO (blue) parasites. Live cells were stained with anti‐EP247 (left), anti‐GPEET 5H3 (right) or no primary antibody (unstained; gray), and analyzed with a Novo‐Cyte flow cytometer. (c) Immunofluorescence microscopy of parental (upper panels) and TbGPI14 KO (lower panels) cells co‐stained with anti‐EP247 and anti‐BiP antibodies. The images selected are representative of the respective cell populations. Scale bar = 5 µm. (d) Anti‐EP and anti‐GPEET immunoblots of whole‐cell protein from parental (P) and TbGPI14 KO cells. Protein from TbGPI13 KO trypanosomes (Verchère et al., 2021) was used as a positive control for defective GPI synthesis. Anti‐aldolase was used as the loading control. Molecular mass markers (in kDa) are indicated in the left margin

TbGPI14 is a 430 amino acid protein sharing 30% identity with its mammalian homolog PIG‐M, and containing the DxD motif that is found in many families of glycosyltransferases (Maeda et al., 2001). TbGPI14 appears as a ~40 kDa band on SDS‐PAGE, migrating faster than expected based on its predicted molecular weight of 49 kDa, consistent with the gel shift commonly observed for membrane proteins (Rath et al., 2009). To study the GPI‐MT‐I function of TbGPI14, we created TbGPI14 null procyclic form *T. brucei* using CRISPR‐Cas9‐targeted gene replacement (with hygromycin and geneticin resistance genes) in the Cas9‐expressing SmOx P9 cell line (Beneke et al., 2017). Successful deletion of both *TbGPI14* alleles was confirmed by a series of PCR reactions using combinations of primers specific to the *TbGPI14* ORF, *TbGPI14* UTRs, and the hygromycin or geneticin resistance gene ORFs (Figure S1). The TbGPI14 null parasites were viable but grew almost 2× more slowly than the parental cells (Figure 1a). Reduced growth in culture has also been reported in *T. brucei* procyclic forms lacking other GPI synthesis enzymes (Hong et al., 2006; Jenni et al., 2021; Nagamune et al., 2000, 2003; Verchère et al., 2021).

### 2.2 TbGPI14 is required for GPI‐anchoring and cell surface expression of procyclins

The surface of procyclic form *T. brucei* cells is densely coated with GPI‐anchored EP and GPEET procyclin proteins (Pays & Nolan, 1998). We used multiple methods to demonstrate that deletion of TbGPI14 leads to a complete loss of GPI‐anchored EP and GPEET procyclins, and a resulting absence of procyclins on the cell surface (Figure 1b–d). Flow cytometry analysis of live trypanosomes stained with anti‐EP or anti‐GPEET antibodies revealed a complete absence of EP and GPEET on the surface of TbGPI14 null cells (Figure 1b). Consistent with this, immunofluorescence microscopy (Figure 1c) showed that whereas parental cells had a clear cell surface pattern of EP labeling, EP was not detected on the surface of TbGPI14 null trypanosomes, but instead appeared to accumulate in the ER as revealed by colocalization with the ER marker BiP. This latter finding differs from a previous study of TbGPI12 null trypanosomes, which found non‐GPI‐anchored EP to accumulate largely in the lysosome (Güther et al., 2009). To demonstrate that in the absence of TbGPI14 EP and GPEET procyclins are still synthesized, but not GPI‐anchored, we carried out anti‐procyclin immunoblotting of SDS‐PAGE‐separated whole‐cell protein from parental and TbGPI14 null parasites (Figure 1d). The results show that in parental cells, anti‐EP247 antibody detected a single band of mature GPI‐anchored EP procyclin at 38–40 kDa, whereas in TbGPI14 null cells, EP was detected as a low molecular mass non‐GPI‐anchored precursor (Figure 1d). Similarly, anti‐GPEET K1 antibody detected GPI‐anchored GPEET at approximately 18 kDa, and a smaller amount of non‐GPI‐anchored GPEET precursor in parental cells, but only the lower molecular mass GPEET precursors were present in TbGPI14 null cells. The anti‐GPEET 5H3 antibody, which only recognizes the mature phosphorylated form of GPEET (Bütikofer et al., 1999), revealed a single broad band at 30–35 kDa in parental cells but showed no reactivity against TbGPI14 null cells. Because GPEET phosphorylation occurs after exiting from the ER (Bütikofer et al., 1999), and therefore, only after GPI attachment, GPEET is not detected by anti‐GPEET 5H3 antibody in TbGPI14 null cells. Together these findings demonstrate that TbGPI14 is required for GPI‐anchoring of EP and GPEET procyclins.

### 2.3 TbGPI14 is required for GPI synthesis

**FIGURE 2:**
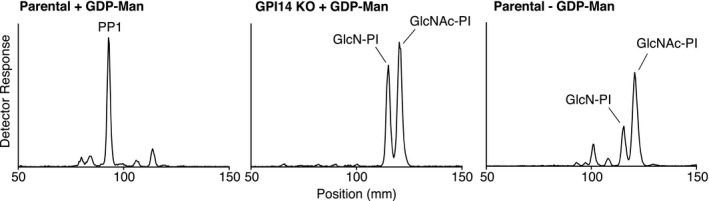
TbGPI14 is required for GPI synthesis in vitro. Membranes from parental (left, right) and TbGPI14 KO (center) parasites were labeled with UDP‐[^3^H]GlcNAc in the presence of tunicamycin, with or without GDP‐mannose as indicated. GPIs were extracted, separated by TLC, and the positions of labeled lipids were detected with a radioisotope scanner. The positions of PP1, GlcN‐PI, and GlcNAc‐PI are indicated. Positions of TLC origin: 20 mm, and front: 178 mm, have been cropped from all traces

To further validate the function of TbGPI14 in *T. brucei*, we used a cell‐free system (Field et al., 1992; Masterson et al., 1989) to analyze GPI synthesis in parental and TbGPI14 null membranes (Figure 2). Membranes were incubated with UDP‐[^3^H]GlcNAc and non‐radioactive GDP‐mannose in the presence of tunicamycin to inhibit [^3^H]GlcNAc‐labelling of dolichol‐linked oligosaccharide precursors of protein *N*‐glycosylation. Thin‐layer chromatography (TLC) analysis of [^3^H]GlcNAc‐labelled GPI lipids revealed PP1, the complete GPI precursor (EtN*P*‐Man_3_GlcN‐(acyl)PI), to be the major product formed by parental membranes (Figure 2, left). In contrast, no PP1 was formed in membranes lacking TbGPI14, instead, we noticed the accumulation of the nonmannosylated GPI precursors [^3^H]GlcN‐PI and [^3^H]GlcNAc‐PI (Figure 2, center); the identities of which were confirmed by comparison with similar extracts from parental membranes (Figure 2, right). A small amount of mannosylated GlcN‐PI is detected in parental membranes in the absence of GDP‐mannose, likely because of the dolichyl‐phosphate‐mannose present in the membrane preparations. Consistent with this result, TbGPI14 null membranes do not produce any mannose‐labeled GPI lipid when incubated with GDP‐[^3^H]mannose in the presence of UDP‐GlcNAc (Figure S2).

### 2.4 Identification of a putative Pbn1 homolog in *T. brucei*

**FIGURE 3:**
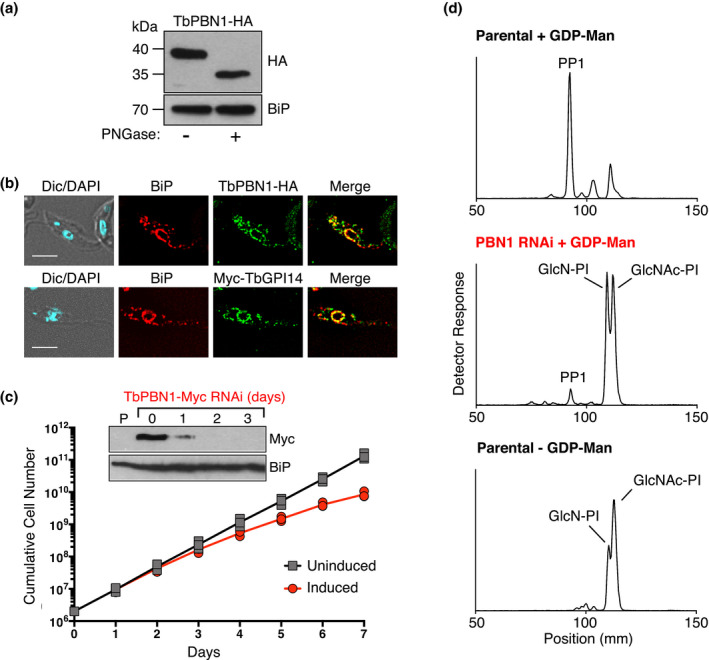
Characterization of TbPBN1. (a) TbPBN1‐HA‐expressing cells were lysed and treated with peptide N‐glycosidase F (PNGase), and the resulting protein extracts were separated by SDS‐PAGE and immunoblotted using anti‐HA (top) and anti‐BiP (loading control, bottom) antibodies. (b) Immunofluorescence microscopy of parasites expressing TbPBN1‐HA (upper panel) or Myc‐TbGPI14 (lower panel), fixed and stained with antibodies against the HA or Myc epitope, together with anti‐BiP (luminal ER marker) antibody. Scale bar = 5 µm. (c) Growth of TbPBN1‐Myc RNAi parasites in the absence (black) or presence (red) of tetracycline. Data points are from two independent experiments. Inset, immunoblot analysis of total cell protein taken prior to RNAi induction (day 0) and after 1, 2, and 3 days of RNAi using anti‐cMyc (TbPBN1) and anti‐BiP (loading control) antibodies. P = Parental. (d) TLC analysis of [^3^H]‐labeled GPI lipids extracted from parental (top, bottom) and TbPBN1‐depleted (middle) membranes incubated with UDP‐[^3^H]GlcNAc in the presence (top, middle) or absence (bottom) of exogenous GDP‐mannose. TbPBN1‐depleted membranes were prepared from TbPBN1‐Myc RNAi cells grown in the presence of tetracycline for 3 days. Positions of TLC origin: 20 mm, and front: 173 mm, have been cropped from all traces

A homolog of the GPI‐MT‐I subunit Pbn1/PIG‐X has not been identified in the *T. brucei* genome. Using remote homology detection searches with *Saccharomyces* *cerevisiae* Pbn1 and mammalian PIG‐X, we identified *TbPBN1* (Tb927.7.5710) as a potential *T. brucei* Pbn1/PIG‐X homolog. *TbPBN1* encodes a 298 amino acid protein with a predicted N‐terminal signal sequence and a single transmembrane domain near its C‐terminus. Sequence alignments show that TbPBN1 shares 15% and 11% amino acid identity with human PIG‐X and *S. cerevisiae* Pbn1, respectively, but that the single C‐terminal transmembrane domain of TbPBN1 is a conserved feature of Pbn1/PIG‐X proteins (Figure S3). Yeast Pbn1 is a much larger protein (416 amino acids) and also shares only 10% identity with its human homolog. Analysis by SDS‐PAGE and immunoblotting of TbPBN1 tagged in situ with a C‐terminal 3xHA epitope (TbPBN1‐HA) revealed a single band with an apparent molecular mass of 40 kDa (Figure 3a). Treatment with peptide‐*N*‐glycosidase F reduced the molecular mass of TbPBN1‐HA to 35 kDa (Figure 3a), indicating that TbPBN1 is *N*‐glycosylated, similar to its mammalian and yeast homologs (Ashida et al., 2005; Naik & Jones, 1998). Mammal and yeast PIG‐X/Pbn1 are found in the ER (Ashida et al., 2005; Naik & Jones, 1998). Using immunofluorescence microscopy, we found that TbPBN1‐HA colocalizes with the ER‐resident protein BiP, predominantly in the perinuclear region of the ER (Figure 3b). A similar localization was also observed for N‐terminally 3xcMyc‐tagged TbGPI14 (Myc‐TbGPI14) (Figure 3b), indicating that as expected both TbPBN1 and TbGPI14 co‐localize in the ER.

### 2.5 TbPBN1 is required for GPI‐MT‐I function

To determine whether TbPBN1 is required for GPI‐MT‐I function, we generated *T. brucei* procyclic forms depleted of TbPBN1. As multiple attempts to generate TbPBN1 null parasites using sequential gene replacement in CRISPR‐capable procyclic forms were unsuccessful, we chose instead to downregulate TbPBN1 expression using tetracycline‐inducible RNAi. To achieve maximal TbPBN1 depletion and allow simple monitoring of TbPBN1 protein levels, we transfected a tetracycline‐inducible *TbPBN1* RNAi construct into a *TbPBN1* single knockout cell line in which the remaining *TbPBN1* allele was 3xcMyc‐tagged (*ΔTbpbn1::HYG/TbPBN1::3cMyc*) (TbPBN1‐Myc). Knockdown of TbPBN1‐Myc was induced by tetracycline addition and the level of TbPBN1‐Myc depletion was analyzed by SDS‐PAGE and immunoblotting. The results show that TbPBN1‐Myc is severely depleted 24 hr after the addition of tetracycline and completely undetectable after 48 hr (Figure 3c). Depletion of TbPBN1 caused a reduction in parasite growth starting at day 3 of RNAi induction (Figure 3c). To determine if depletion of TbPBN1 caused a reduction in GPI‐MT‐I activity, we analyzed GPI synthesis using membranes prepared on day 3 of TbPBN1 RNAi induction. As with TbGPI14 null membranes (see Figure 2), TbPBN1‐depleted membranes accumulated [^3^H]GlcNAc‐PI and [^3^H]GlcN‐PI when incubated with UDP‐[^3^H]GlcNAc and GDP‐mannose, whereas parental cells produced [^3^H]‐labeled PP1 (Figure 3d), indicating that TbPBN1‐depleted membranes have a very low level of GPI‐MT‐I activity. Similarly, TbPBN1‐depleted membranes incubated with GDP‐[^3^H]mannose and UDP‐GlcNAc produced significantly less [^3^H]‐labeled GPI products than the parental membranes (Figure S2). The small amount of [^3^H]‐labeled PP1 produced by TbPBN1‐depleted membranes is likely due to incomplete TbPBN1‐Myc depletion by RNAi.

### 2.6 Loss of GPI‐anchored procyclins after TbPBN1 depletion

**FIGURE 4:**
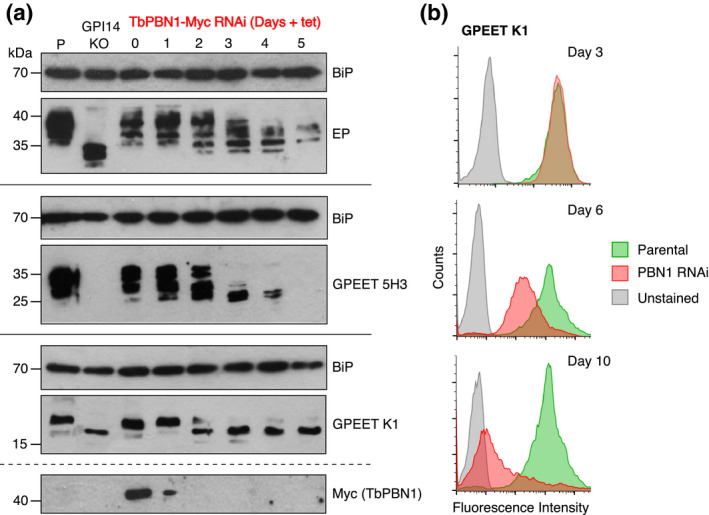
Loss of GPI‐anchored procyclins after TbPBN1‐Myc RNAi. (a) Total cell protein from parental (Lane P), GPI14 KO (Lane KO), and TbPBN1 RNAi parasites after 0–5 days of RNAi induction (Lanes 0–5) were separated by SDS‐PAGE and immunoblotted with anti‐EP247 (top), anti‐GPEET 5H3 (middle), anti‐GPEET K1 (bottom), or anti‐cMyc (RNAi control to monitor TbPBN1‐Myc depletion, very bottom) antibodies. Anti‐BiP was used as a loading control for each immunoblot; the bottom two panels are separated by a dashed line as they have the same loading control. (b) Flow cytometry of live parental (green) and TbPBN1 RNAi (red) parasites stained with anti‐GPEET K1 antibody. Parasites stained only with secondary antibodies (unstained; gray) were used as a negative control. The number of days of induction of TbPBN1 RNAi is indicated

To study the effect of TbPBN1 depletion on GPI‐anchoring and cell surface procyclin expression, parental parasites and TbPBN1‐Myc cells during RNAi were analyzed by SDS‐PAGE/immunoblotting and flow cytometry using anti‐procyclin antibodies. Immunoblot analyses using anti‐EP247 (Figure 4a, top) and anti‐GPEET 5H3 (Figure 4a, middle) antibodies revealed a gradual reduction in molecular mass of both EP and GPEET procyclin, starting around day 2 after induction of TbPBN1 depletion. This likely represents a loss of mature GPI‐anchored procyclins and concurrent accumulation of unanchored procyclin precursors. Consistent with the results obtained for TbGPI14 null parasites (see Figure 1d), the GPEET 5H3 signal was completely lost by day 5 of TbPBN1 depletion. However, unlike in TbGPI14 null parasites, the levels of unanchored EP procyclin also appeared to decline during TbPBN1 depletion. Complete loss of EP has also been reported after depletion of TbGPI8, the catalytic subunit of the transamidase required for attachment of the GPI anchor to procyclins (Knüsel, S. and Roditi, I., personal communication). In addition, immunoblotting using anti‐GPEET K1 antibody also revealed a gradual loss of GPI‐anchored GPEET (Figure 4a, bottom). Prior to TbPBN1 depletion, GPI‐anchored GPEET, at approximately 20 kDa, was the major product detected by anti‐GPEET K1 antibody, with only a small amount of lower molecular weight unanchored GPEET detected at 16–18 kDa. However, by day 2 of TbPBN1 depletion, unanchored GPEET was the most abundant form, and from day 3 on GPI‐anchored GPEET was barely detectable. Similarly, flow cytometry analysis of live parasites stained with anti‐GPEET K1 antibody revealed a gradual loss of GPEET procyclin from the cell surface after TbPBN1 RNAi induction (Figure 4b).

### 2.7 TbGPI14 and TbPBN1 interact

**FIGURE 5:**
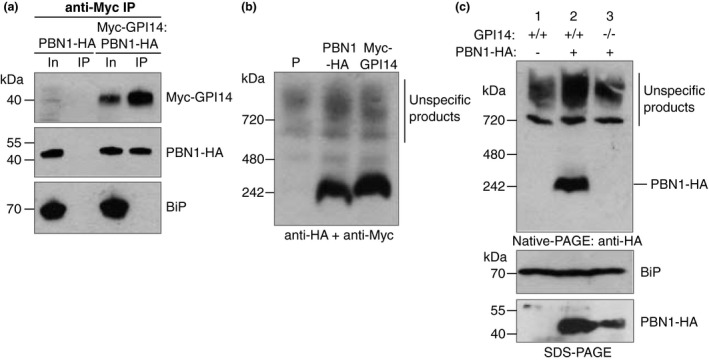
Interaction of TbPBN1 and TbGPI14. (a) Co‐immunoprecipitation of Myc‐TbGPI14 with TbPBN1‐HA. Parasites expressing Myc‐TbGPI14 and TbPBN1‐HA, or TbPBN1‐HA only, were lysed with 1% DDM followed by immunoprecipitation with anti‐cMyc beads. Precipitated proteins were separated by SDS‐PAGE and immunoblotted with anti‐cMyc (top), anti‐HA (middle), or anti‐BiP (bottom; control) antibodies. In = Input, 2% of the DDM lysate applied to the anti‐cMyc beads, IP = 10% of the protein remaining on the anti‐cMyc beads after extensive washing. (b) Native PAGE analysis of proteins from cells expressing either TbPBN1‐HA, Myc‐TbGPI14, or no tag (P), immunoblotted sequentially with anti‐HA and anti‐cMyc antibodies. Under these conditions, the anti‐HA antibody consistently produced nonspecific products above 720 kDa, as labeled on the blot. (c) Top panel, native PAGE of proteins from cells expressing TbPBN1‐HA in parental (Lane 2) or TbGPI14 null (Lane 3) parasites, and from untagged parental cells (Lane 1), immunoblotted with anti‐HA antibody. Bottom panels, SDS‐PAGE of proteins from the same cell lines immunoblotted with anti‐HA or anti‐BiP (loading control) antibodies. Equal numbers of cell equivalents were loaded in each lane

Mammalian PIG‐M and PIG‐X were previously shown to interact in co‐immunoprecipitation experiments (Ashida et al., 2005). To test whether TbGPI14 and TbPBN1 also interact, we created a double‐tagged procyclic form cell line stably expressing N‐terminally 3xcMyc‐tagged TbGPI14 (Myc‐TbGPI14) and C‐terminally 3xHA‐tagged TbPBN1 (TbPBN1‐HA) (*3xcMyc::TbGpi14/TbPbn1::3xHA*). From these double‐tagged parasites, lysed with 1% *n*‐dodecyl‐β‐d‐maltoside (DDM), Myc‐TbGPI14 was immunoprecipitated with anti‐cMyc antibody and the presence of coprecipitated TbPBN1‐HA was assessed by SDS‐PAGE and immunoblotting. We found that TbPBN1‐HA coprecipitated with Myc‐TbGPI14, whereas the ER protein BiP, selected as a control for nonspecific interactions, did not (Figure 5a), indicating that TbGPI14 and TbPBN1 interact with each other specifically.

To characterize the TbGPI14–TbPBN1 complex further, parasites expressing Myc‐TbGPI14 or TbPBN1‐HA were analyzed by native PAGE followed by anti‐HA and anti‐cMyc immunoblotting. Under these conditions, both Myc‐TbGPI14 and TbPBN1‐HA migrated at approximately 240 kDa (Figure 5b), higher than would be expected for a TbGPI14‐TbPBN1 dimer, which should run at ~80 kDa. This suggests that the proteins may form a higher‐order complex. The 240 kDa TbPBN1‐HA band was absent on native PAGE when expressed in a TbGPI14 KO background (Figure 5c, top) despite being detectable after SDS‐PAGE, although at a lower level of expression than in TbGPI14‐expressing cells (Figure 5c, bottom). Reduced TbPBN1‐HA expression in the TbGPI14 KO background suggests that TbGPI14 affects TbPBN1 stability, as seen with the mammalian homologs (Ashida et al., 2005). Taken together, these results support the presence of a higher‐order complex containing TbGPI14 and TbPBN1.

### 2.8 Yeast Gpi14 does not complement TbGPI14 deletion

**FIGURE 6:**
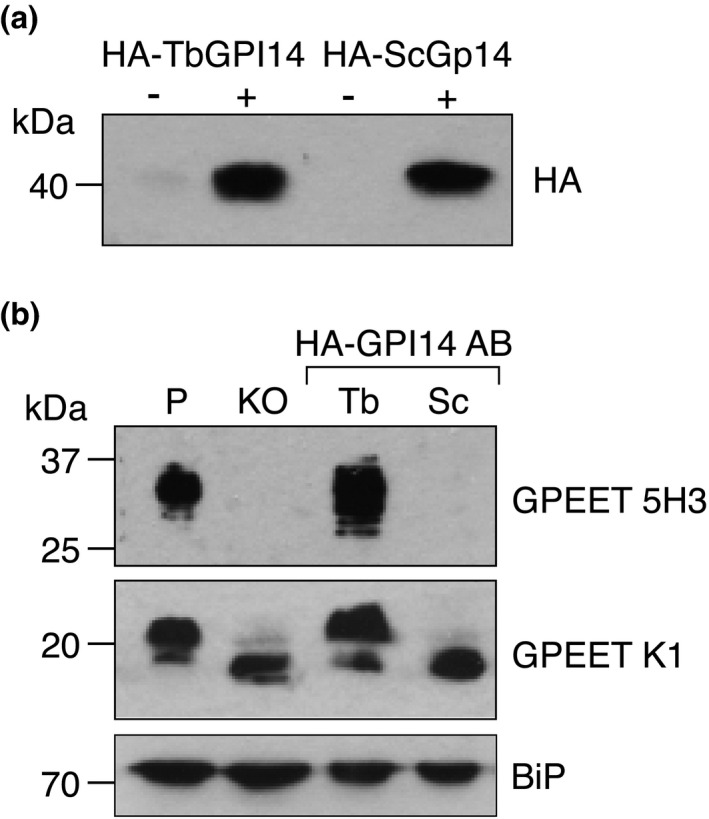
HA‐ScGpi14 does not restore GPI‐MT‐I function in TbGPI14 KO parasites. (a) HA‐GPI14 add back cell lines were grown in the presence (+) or absence (−) of tetracycline and HA‐GPI14 protein enriched by anti‐HA immunoprecipitation before detection by SDS‐PAGE and immunoblotting with anti‐HA antibody. (b) Immunoblot of protein from parental (P), TbGPI14 KO (KO), and HA‐TbGPI14 (Tb) and HA‐ScGPI14 (Sc) add back parasites with anti‐GPEET 5H3 (top), anti‐GPEET K1 (middle), or anti‐BiP (bottom; loading control) antibodies. Add back parasites were maintained in the presence of tetracycline to induce HA‐GPI14 expression

It has been previously shown that functional interactions between yeast Gpi14 and Pbn1, and mammalian PIG‐M and PIG‐X, respectively, are species specific and essential for GPI‐MT‐I activity. Mammalian PIG‐M only complemented Gpi14‐deficient yeast when mammalian PIG‐X was present (Kim et al., 2007). Similarly, yeast Pbn1 restored GPI‐MT‐I function in PIG‐X‐deficient mammalian cells only when coexpressed with yeast Gpi14 (Ashida et al., 2005). In addition, it was shown that TbGPI14 was unable to complement Gpi14‐deficient yeast (Kim et al., 2007), however, it is not known whether this was due to the absence of TbPBN1 or the different substrate specificities of yeast and *T. brucei* GPI‐MT‐I. To test whether yeast Gpi14 can form a functional GPI‐MT‐I complex with trypanosome PBN1, we expressed N‐terminally 2xHA‐tagged TbGPI14 or *S. cerevisiae* Gpi14 (ScGpi14) from a tetracycline‐inducible plasmid in TbGPI14 KO parasites. Both proteins were induced by tetracycline addition and detectable after enrichment by anti‐HA immunoprecipitation (Figure 6a). As expected, reexpression of HA‐TbGPI14 in the TbGPI14 KO cell line restored GPI‐MT‐I function, as demonstrated by the restoration of GPI‐anchored GPEET detected by anti‐GPEET K1 and 5H3 immunoblots. In contrast, equal levels of HA‐ScGpi14 expression did not restore GPI‐MT‐I function in TbGPI14 KO cells (Figure 6b). Anti‐GPEET K1 immunoblotting showed the return of the higher molecular weight GPI‐anchored GPEET in TbGPI14 KO parasites expressing HA‐TbGPI14 but not HA‐ScGpi14. Similarly, phosphorylated GPEET (and therefore GPI‐anchored), detected by anti‐GPEET 5H3 antibody, was only present in the HA‐TbGPI14 add back. Thus, yeast Gpi14 cannot functionally replace TbGPI14.

## 3 CONCLUDING REMARKS

GPI‐MT‐I is a heteromeric protein complex that catalyzes the first mannosylation reaction of GPI biosynthesis, transferring mannose from dolichyl‐phosphate‐mannose to GlcN‐PI or GlcN‐(acyl)PI. Extending studies in mammals and yeast, we now report that *T. brucei* GPI‐MT‐I also consists of two essential subunits related to mammalian/yeast PIG‐M/Gpi14 and PIG‐X/Pbn1 (Ashida et al., 2005; Kim et al., 2007). By creating a TbGPI14 knockout procyclic form *T. brucei* cell line, and depleting TbPBN1 using RNAi, we show that both proteins are essential for GPI synthesis in cells as well as in a derived cell‐free system. We additionally show that the TbGPI14 and TbPBN1 proteins colocalize in the (perinuclear region of the) ER and physically interact, and that TbPBN1 may be stabilized by the presence of TbGPI14, as previously shown with mammalian PIG‐M and PIG‐X (Ashida et al., 2005). Native PAGE analysis showed that both TbGPI14 and TbPBN1 migrate at approximately 240 kDa under nondenaturing conditions. As a TbGPI14‐TbPBN1 dimer would have a predicted mass of approximately 80 kDa, this suggests the presence of a higher‐order complex made up of, or containing, TbGPI14–TbPBN1 dimers. Whether the complex contains other proteins is a subject for future work.

The functional interaction of GPI‐MT‐I subunits is known to be species specific. Mammalian PIG‐M can function in yeast cells, but only in the presence of mammalian PIG‐X, and likewise, yeast Gpi14 can rescue mammalian PIG‐M deletion when yeast Pbn1 is present (Ashida et al., 2005; Kim et al., 2007). It was previously shown that *T. brucei* GPI14 cannot rescue Gpi14‐depleted yeast (Kim et al., 2007), and our data now suggest that this was due to the absence of TbPBN1. Similarly, GPI14/PIG‐M from related protozoa *Trypanosoma cruzi* and *Plasmodium falciparum* do not complement Gpi14‐depletion in yeast (Cardoso et al., 2013; Kim et al., 2007), suggesting that these proteins also possess as yet unidentified GPI‐MT‐I accessory subunits. Here we show that *S. cerevisiae* Gpi14 does not restore GPI‐MT‐I function in TbGPI14 KO *T. brucei*, consistent with the species specificity of the Gpi14‐Pbn1 functional association. However, though mammalian GPI‐MT‐I can mannosylate GlcN‐PI (Murakami et al., 2003), yeast and mammalian GPI‐MT‐I favor GlcN‐(acyl)PI as a substrate (Doerrler et al., 1996; Murakami et al., 2003). It was even shown that synthetic GlcN‐(2‐*O*‐Me)PI, for which inositol acylation is blocked, is a substrate for *T. brucei* GPI‐MT‐I, but not for mammalian GPI‐MT‐I, in cell‐free assays (Smith et al., 1997). *T. brucei* cannot acylate the inositol of non‐mannosylated GlcN‐PI (Güther & Ferguson, 1995), therefore, GlcN‐(acyl)PI will not be produced in TbGPI14 null trypanosomes. Consequently, it is possible that even a complete yeast GPI‐MT‐I (Gpi14 + Pbn1) would be unable to complement TbGPI14 deletion. Similarly, it remains unclear whether *T. brucei* GPI‐MT‐I would be able to mannosylate GlcN‐(acyl)PI in yeast or mammalian systems, and further complementation studies could now address these questions.

Whereas PIG‐M/Gpi14/TbGPI14 with its functionally important DxD motif is clearly the catalytic subunit of GPI‐MT‐I, the function of the noncatalytic PIG‐X/Pbn1/TbPBN1 subunit is not clear. TbGPI14 regulates the expression of TbPBN1 as steady‐state levels of the latter are lower in the absence of the former, and in mammalian cells, PIG‐M expression depends on PIG‐X. Perhaps, TbPBN1 is important in recruiting the dolichyl‐phosphate mannose substrate to the enzyme, or functions to incorporate GPI‐MT‐I into a GPI biosynthetic metabolon, the latter consistent with our discovery of a higher‐order 240 kDa complex containing TbGPI14 and TbPBN1. Further work will be needed to investigate these possibilities. Yeast Pbn1 is also known to act as a chaperone required for proper folding and processing of GPI‐anchored and unanchored ER proteins (Subramanian et al., 2006). It remains to be seen if such chaperone functions are associated with TbPBN1, or whether the much larger size of yeast Pbn1 (416aa [Sc] vs. 298aa [Tb]) can account for these extra functions. The nonessentiality of TbPBN1, and GPI synthesis in general, in procyclic form *T. brucei* makes this an ideal system to study the role of Pbn1 in GPI‐MT‐I.

## 4 METHODS

### 4.1 Culture and transfection of *T. brucei* cells

Two procyclic form *T. brucei* cell lines were used in this study. SmOx P9 is a TREU927/4 cell line modified to express Cas9 and T7 RNA polymerase (Beneke et al., 2017). Cas9/TetR cells are an early procyclic form Lister 427‐derived cell line that express Cas9, T7RNAP, and the tetracycline repressor (TetR) and was a kind gift from Isabel Roditi (University of Bern, Switzerland) (modified from Shaw et al., 2019). All cells were cultured at 27°C in SDM‐79 medium supplemented with 10% fetal bovine serum and the appropriate antibiotics: 10 µg/ml blasticidin and 1 µg/ml puromycin for SmOx P9, and 10 µg/ml blasticidin and 130 µg/ml NTC for Cas9/TetR cells. Transfections were carried out by electroporating 3 × 10^7^ cells with donor DNA ± sgRNA templates in 1xTbBSF buffer (90 mM sodium phosphate, 5 mM potassium chloride, 0.15 mM calcium chloride, 50 mM HEPES, pH 7.3), total volume 100 µl, using the Amaxa Nucleofector 4D (Lonza), program FI‐115. Clonal populations of successfully modified trypanosomes were obtained by clonal dilution in the presence of the appropriate selecting antibiotic.

### 4.2 Construction of *T. brucei* cell lines

TbGPI14 null parasites (*ΔTbgpi14::HYG/ΔTbgpi14::G418*) were generated in SmOx p9 cells utilizing the CRISPR‐Cas9 system as previously described (Beneke et al., 2017). Geneticin and hygromycin resistance cassettes flanked by 30 bp of *TbGPI14* 5′ and 3′ UTR sequences were generated by PCR from pPOTv7 plasmids using primers 1 and 2 (Table S1). Gene‐specific 5′ and 3′ short guide RNA (sgRNA) templates, consisting of a T7 RNA polymerase promoter sequence and a short sequence of homology with the *TbGPI14* 5′ or 3′ UTR, were generated by annealing TbGPI14‐specific primers (Table S1, primers 3 and 4) with the G00 sgRNA scaffold primer. *TbGpi14* gene replacement was achieved by co‐transfection of the gene‐specific geneticin or hygromycin cassette and both 5′ and 3′ sgRNA templates, and selection with 25 µg/ml geneticin and 50 µg/ml hygromycin.

In‐frame tagging of endogenous *TbGPI14* and *TbPBN1* genes was also achieved using the CRISPR‐Cas9 system as described previously (Beneke et al., 2017). Donor DNA containing a resistance gene and the tag sequence flanked by 30 bp regions of homology specific to the target gene was amplified from the appropriate pMOTag plasmid (Oberholzer et al., 2006) using gene‐specific primers (TbGPI14: primers 6 + 7, TbPBN1: primers 8 + 9, Table S1). Gene tagging was then achieved by cotransfecting this donor DNA with either 5′ (for N‐terminal tagging) or 3′ (for C‐terminal tagging) gene‐specific sgRNA, prepared by annealing either primer 3 (TbGPI14) or 10 (TbPBN1) with the G00 primer (Table S1). Successful integration of donor DNA was selected for with combinations of 25 µg/ml geneticin (TbPBN1‐HA), 2 µg/ml puromycin (TbPBN1‐Myc), or 50 µg/ml hygromycin (Myc‐TbGPI14).

TbPBN1 RNAi was performed in Cas9/TetR cells using the tetracycline‐inducible stem‐loop pALC14 vector (Bochud‐Allemann & Schneider, 2002). pALC14‐TbPBN1 was created by amplifying an approximately 400 bp fragment of *TbPBN1*, in sense and antisense orientation, from *T. brucei* genomic DNA, using primer pairs 14 + 15 and 16 + 17, respectively (Table S1). The resulting products were sequentially cloned into pALC14 on either side of a stuffer region using *HindIII* + *XbaI* and *XhoI* + *BamHI*. To create the TbPBN1 RNAi cell line, first a *TbPBN1* single‐allele deletion (*ΔTbpbn1::HYG/TbPBN1*) was made by gene replacement with a hygromycin resistance cassette as described above. The gene replacement cassette was amplified from pPOTv7 using primers 11 + 12, and 5′ and 3′ sgRNA was prepared by annealing primers 13 or 10 with the G00 primer, respectively (Table S1). Next, the remaining *TbPBN1* allele was C‐terminally 3xcMyc tagged (*ΔTbpbn1::HYG/TbPBN1::3cMyc*) as described above. Finally, 10 µg of *NotI*‐linearized pALC‐TbPBN1 was transfected into this cell line and successful transformants were selected with 4 µg/ml phleomycin. *TbPBN1* RNAi was induced with 1.5 µg/ml tetracycline and TbPBN1‐Myc depletion was confirmed by anti‐cMyc immunoblotting.

For GPI14 reexpression cell lines, full‐length *TbGPI14* or *ScGpi14* sequences were amplified from the corresponding genomic DNA (primers 23 + 24 and 25 + 26, respectively, Table S1) and ligated into pMS‐HA, a tetracycline‐inducible expression vector (Serricchio & Bütikofer, 2013) modified to allow N‐terminal 2xHA‐tagging, using *NdeI* and *BamHI*. *NotI*‐linearized pMS‐HA‐TbGPI14 or pMS‐HA‐ScGPI14 (10 µg) were transfected into TbGPI14 null cells and clones selected for with 4 µg/ml phleomycin. Expression HA‐TbGPI14 or HA‐ScGpi14 was induced by the addition of 1.5 µg/ml tetracycline to the growth medium.

### 4.3 Diagnostic PCR

Deletion of the *TbGPI14* gene was confirmed by PCR analysis. Genomic DNA was extracted from 1 to 2 × 10^7^ parasites washed once with TE (10 mM Tris, 0.1 mM EDTA, pH 8.0) and lysed with 0.1% SDS in 100 µl TE, at 55°C for 10 min. Potassium acetate (3M/5M), 30 µl, was then added and the samples were incubated on ice for 5 min before centrifugation at 17,000 × *g*. The supernatant was transferred to a fresh tube and DNA precipitated by adding 2× volume of 100% ethanol. The DNA was pelleted at 17,000 × *g* for 10 min, 4°C, washed once with 70% ethanol, and finally suspended in H_2_O. Four different PCRs were carried out using combinations of primers specific to the *TbGPI14* 5′ and 3′UTRs, *TbGPI14* ORF, and the genetic and hygromycin resistance gene ORFs (Figure S1, Table S1, primers 18–22). The PCR products were then run on a 1% agarose gel to check for the presence or absence of the expected products.

### 4.4 Growth curve

*TbGPI14* null and parental SmOx p9 cell lines were diluted to 2 × 10^6^ cells/ml in a volume of 5 ml on day 0, with three replicates per cell line. Cells were then counted every 24 hr and always diluted back to 2 × 10^6^ cells/ml regardless of growth. For the TbPBN1 RNAi growth curve, three separate TbPBN1 RNAi cell lines were used. Each cell line was divided into two culture flasks at 2 × 10^6^ cells/ml in 5 ml SDM‐79, one with 1.5 µg/ml tetracycline and the other without. Every culture was then counted every 24 hr and always diluted in fresh media (with or without tetracycline) back to 2 × 10^6^ cells/ml.

### 4.5 PNGase F treatment

Trypanosomes (1 × 10^7^ cells) were harvested, washed twice with Tris‐buffered saline (TBS: 10 mM Tris–HCl pH 7.5, 144 mM NaCl), and lysed in 20 µl 1× denaturing buffer (0.5% SDS, 40 mM dithiothreitol) by boiling at 100°C for 10 min. Lysed cells were treated with 2 µl peptide‐*N*‐glycosidase F (PNGase F, New England Biolabs) at 37°C for 1 hr in a buffer containing 1% NP‐40, total volume 30 µl, following the protocol, and using buffers, provided by the manufacturer (New England Biolabs). Protein samples were prepared for SDS‐PAGE analysis by adding 10 µl 4× SDS sample buffer to the PNGase‐treated lysates and heating at 55°C for 10 min.

### 4.6 Cell‐free GPI synthesis

Trypanosome membranes for cell‐free GPI synthesis were prepared by hypotonic lysis as previously described (Masterson et al., 1989), except that tunicamycin was omitted from cultures prior to lysis. Briefly, 1 × 10^9^ mid‐log cells were harvested, washed once with ice‐cold buffer 1 (50 mM Na‐Bicine [pH 8.0], 5 mM KCI, 50 mM NaCI, 10 mg/ml glucose, 1 mg/ml bovine serum albumin), and lysed hypotonically in 1 ml water containing 0.1 mM TLCK and 1 µg/ml leupeptin, on ice for 5 min. An equal volume of HKMTLG buffer (100 mM Na‐HEPES (pH 7.4), 50 mM KCI, 10 mM MgCl_2_, 0.1 mM TLCK, 1 µg/ml leupeptin, 20% glycerol) was then added, to a final concentration of 5 x 10^8^ cells/ml, and the lysate aliquoted, snap‐frozen and stored at −70°C.

Membrane aliquots were thawed, washed twice with ice‐cold HKMTL buffer (50 mM Na‐HEPES pH7.4, 25 mM KCl, 5 mM MgCl_2_, 0.1 mM TLCK, 1 µg/ml leupeptin) and resuspended in 2× incorporation buffer (50 mM Na‐HEPES pH 7.4, 25 mM KCl, 5 mM MgCl_2_, 0.1 mM TLCK, 1 µg/ml leupeptin, 10 mM MnCl_2_, 1 µg/ml tunicamycin) at 1.66 × 10^9^ cells/ml. For each reaction, 30 µl of prepared membranes (5 × 10^7^ cells) was added to an equal volume of water containing 0.5 µCi UDP‐[^3^H]GlcNAc, 2 mM ATP, 2 mM dithiothreitol, with or without 2 mM GDP‐mannose, and incubated at 28°C for 30 min. Glycolipids were extracted as previously described (Leal et al., 2004). Briefly, lipids were first extracted by adding 400 µl chloroform/methanol (1:1; v/v), reextracted with a further 250 µl chloroform/methanol/water (10:10:3; v/v/v), and the pooled organic supernatants dried under a N_2_ stream. Lipids were then partitioned twice between water‐saturated *n*‐butanol and butanol‐saturated water (100 µl of each), and the *n*‐butanol phases were collected, pooled, and back‐washed with 200 µl butanol‐saturated water. The final *n*‐butanol phase was dried under a stream of N_2_, resuspended in 30 µl chloroform/methanol/water (10:10:3; v/v/v), and separated by thin‐layer chromatography (TLC) on silica gel 60 plates (Merck) using chloroform/methanol/water (10:10:3; v/v/v) as the solvent system. The positions of radiolabeled lipids were analyzed by scanning the TLC plates with a radioisotope scanner (Berthold Technologies, Regensburg, Switzerland).

### 4.7 Flow cytometry analysis

Procyclin surface expression was analyzed by flow cytometry with live cells. Trypanosomes (5 × 10^6^ cells) were pelleted and resuspended in SDM‐79 containing one of the following primary antibodies: anti‐EP247 (Cedarlane, 1:500 dilution), anti‐GPEET K1 (courtesy of Isabel Roditi, 1:1,000), or anti‐GPEET 5H3 (courtesy of Isabel Roditi, 1:1,000), and incubated for 30 min at 4°C. Cells were washed twice with SDM‐79 then incubated with anti‐rabbit or anti‐mouse AlexaFluor 488 (Invitrogen) secondary antibody (both 1:1,000) in SDM‐79 for 30 min at 4°C. After two more washes with SDM‐79, samples were analyzed with a Novo‐Cyte flow cytometer (ACEA Biosciences).

### 4.8 Immunofluorescence microscopy

Approximately 4 × 10^6^ trypanosomes were pelleted, washed twice with ice‐cold PBS (137 mM NaCl, 2.7 mM KCl, 10 mM Na_2_PO_4_, 2 mM KH_2_PO_4_, pH 7.4), resuspended in PBS, and pipetted onto microscope slides. Cells were left to adhere to the slide, then fixed with 4% paraformaldehyde in PBS for 10 min at room temperature. Fixed cells were washed three times with PBS then permeabilized with 0.2% (w/v) Triton X‐100 in PBS for 15 min. Cells were washed again three times then blocked with 2% (w/v) bovine serum albumin in PBS for 30 min, followed by incubation with the primary antibody in 1% (w/v) bovine serum albumin in PBS for 45 min. Primary antibodies used were rabbit anti‐BiP (a gift from James Bangs, University at Buffalo, Buffalo, NY) diluted 1:2,500, mouse anti‐EP247 diluted 1:2,000, and mouse anti‐HA (clone 16B12, Enzo Life Sciences) and anti‐cMyc (Santa Cruz Biotechnology) both at a 1:200 dilution. Cells were washed three times with PBS before incubation with fluorescent secondary antibodies, Alexa Fluor^®^ goat anti‐mouse 488 (1:2,000, Invitrogen™), and goat anti‐rabbit 594 (1:1,000, Invitrogen™), in 1% bovine serum albumin in PBS for 45 min. After three final washes, coverslips were mounted on dried slides with Vectashield^®^ containing 4',6‐diamidino‐2‐phenylindole (DAPI, Vector Laboratories). Images of stained cells were taken with a Leica DM 16000 B inverted microscope combined with a Leica DFC360 FX camera, and image deconvolution (3D deconvolution) was performed using Leica LAS X software.

### 4.9 SDS‐PAGE and immunoblotting

To prepare protein samples for SDS‐PAGE trypanosomes were pelleted by centrifugation, washed two times with PBS, resuspended in 1× SDS sample buffer (100 mM Tris–HCl, 4% SDS, 20% glycerol, 0.05% bromophenol blue, pH 6.8) and then heated at 55°C for 10 min. Samples (generally 3–5 × 10^6^ cell equivalents) were separated by SDS‐PAGE on 12% polyacrylamide gels under reducing conditions and transferred to nitrocellulose membrane. Membranes were blocked with 5% milk in TBS, and primary and secondary antibodies diluted in 1% milk in TBS + 0.1% Tween‐20 (TBST). Washing of the membranes after antibody incubations were carried out with TBST. The following primary antibodies were used: anti‐EP247 (1:1,500), anti‐GPEET K1 (1:2,500), anti‐GPEET 5H3 (1:5,000), and anti‐aldolase (a gift from Paul Michels, University of Edinburgh, Scotland, 1:180,000). HRP‐conjugated secondary anti‐rabbit and anti‐mouse antibodies (DAKO) were used at 1:1,000 and 1:5,000, respectively, and chemiluminescent detection was carried out using SuperSignal™ Pico West PLUS substrate (ThermoScientific).

### 4.10 Native PAGE

Native PAGE was carried out as previously described (Serricchio & Bütikofer, 2012). Briefly, 1 × 10^8^ cells were harvested, washed once with SBG buffer (150 mM Tris–HCl pH 7.9, 20 mM glucose, 20 mM NaH_2_PO_4_), and lysed by incubation with 0.025% digitonin in SoTE buffer (20 mM Tris–HCl pH 7.5, 600 mM sorbitol, 2 mM EDTA) on ice for 5 min. After centrifugation at 6,000 × *g* for 5 min, the resulting membrane pellet was lysed further with 1.5% digitonin in Buffer A (20 mM Tris–HCl pH 7.2, 15 mM KH_2_PO_4_, 20 mM MgSO_4_, 600 mM sorbitol) for 15 min on ice, the soluble fraction collected by centrifugation at 17,200 × *g* for 30 min and mixed with 1× native loading buffer (0.5% Coomassie Brilliant Blue G250, 50 mM 6‐aminocaproic acid, 10 mM Bis–Tris–HCl pH 7.0). Protein from approximately 3 × 10^7^ cell equivalents were then separated on 4%–13% acrylamide gradient gels under nondenaturing conditions, transferred to nitrocellulose membranes, and detected using HRP‐conjugated anti‐HA (Roche, 1:1,000) or anti‐cMyc (Roche, 1:3,000) antibodies.

## CONFLICT OF INTEREST

The authors declare that they have no conflicts of interest.

## Supporting information

Supplementary Material

## Data Availability

The data that support the findings of this study are available in the figures and supplementary material of this article and/or are available from the authors upon request.
