# Supplementary material for: Identification of TbPBN1 in Trypanosoma brucei reveals a conserved heterodimeric architecture for glycosylphosphatidylinositol‐mannosyltransferase‐I
Source: Mol Microbiol. 2021 Dec 25;117(2):450–61. doi: 10.1111/mmi.14859 (PMC9306709; doi:10.1111/mmi.14859)
Supplement: Supplementary file 1 — Supplementary Material [file MMI-117-450-s001.pdf]

Supplemental figures for:

Identification of TbPBN1 in *Trypanosoma brucei* reveals a conserved heterodimeric architecture for glycosylphosphatidylinositol-mannosyltransferase I

Andrew Cowton[1*], Peter Bütikofer[1], Robert Häner[2], Anant K. Menon[3]

[1] Institute of Biochemistry and Molecular Medicine, University of Bern, Bern, Switzerland

[2] Department of Chemistry, Biochemistry and Pharmaceutical Sciences, University of Bern, Bern, Switzerland

[3] Department of Biochemistry, Weill Cornell Medical College, New York, New York, USA

* Corresponding author.

Correspondence to:
Andrew Cowton, Ph.D.
andrew.cowton@ibmm.unibe.ch

Content:

Figure S1: PCR confirmation of TbGPI14 knockout
Figure S2: Cell free labeling of GPI precursors with GDP-[^{3}H]mannose by TbGPI14 KO and TbPBN1 RNAi membranes
Figure S3: Alignment of the amino acid sequences of TbPBN1 and the human, rat and *S. cerevisiae* homologues
Table S1: List of primers used in this study

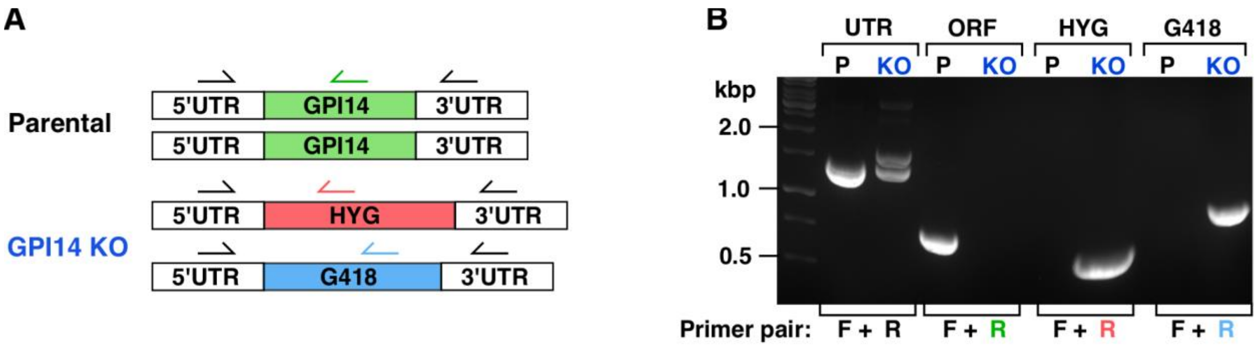

Figure S1: PCR confirmation of TbGPI14 knockout. A) Diagram of the *TbGPI14* locus in parental and knockout parasites and the locations of the primers (half arrows) used to detect replacement of the *TbGPI14* ORF with *Hyg* and *G418* resistance genes. B) PCR products from reactions using primers depicted in A) with genomic DNA extracted from parental (P) and TbGPI14 KO (KO) parasites. F = forward primer, R = reverse primer, and both are colour coded to match the corresponding primers depicted in A).

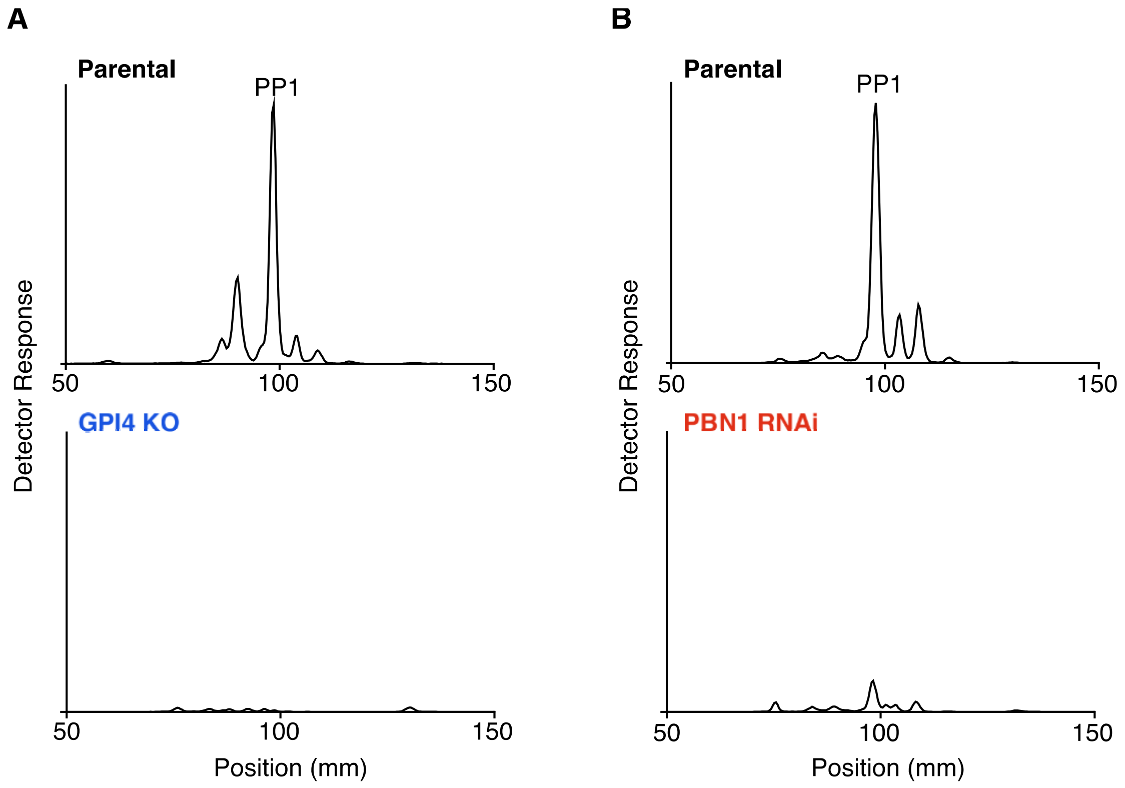

Figure S2: Cell free labeling of GPI precursors with GDP-[^{3}H]mannose by TbGPI14 KO and TbPBN1 RNAi membranes. TLC analysis of GPIs produced by membranes from A) TbGPI14 KO, B) TbPBN1 RNAi, or the corresponding parental (A and B, upper panels) parasites, after incubation with GDP-[^{3}H]mannose and UDP-GlcNAc. The position of the PP1 is indicated, and positions of the origin (20 mm) and front (178 mm) have been cropped from the traces.

```
TbPBN1       ------------------------------------------------------------------------
HsPig-X      ------------------------------------------------------------------------
RnPig-X      ------------------------------------------------------------------------
ScPbn1    1  MVTRHRVTVLYNAPEDIGNHMRQNDTHLTVRGGSGVVLQQRWLLERTGSLDKSFTRITWRPRADLARSLSVI  72

TbPBN1       ------------------------------------------------------------------------
HsPig-X      ------------------------------------------------------------------------
RnPig-X      ------------------------------------------------------------------------
ScPbn1   73  ENELSAGFSVYSNSSDVPERFITNPVYNSFHSEKFDIEQYLPPEVDLNLSWNPEDFTYDISVEPTQIQIVEY  144

TbPBN1    1  -------MFTSTAAYCGSAGSRLFVVALLAFCVVTGTTVSD--ERETVNSSCENPSLHDTH-HW-------  55
HsPig-X   1  ---------MAARVAAVRA-AAWLLLGAATGLTRGPAAAFTAARSDAGIRAMCSEIIL--RQE---------  51
RnPig-X   1  --------------MAASA-LAWLLLWA-AGLVGRLAADISDARFSDGVRATCSEIIL--RQE---------  45
ScPbn1  145  RLLKQGEEFTIARVKDEKLEVGVFFVDASDESDVDIGGIRCNWRMDDGKMERCQKTSLLYKQGHIAYNHSTT  216

TbPBN1   56  ---GFL--GGGYHMQLEVEF--PLIFN-----SVDISLDLPRTFFFDAAELEQLYSIKLQGSKEDITWAYQP  115
HsPig-X  52  ----VL--KDGFHRDLLIKVKFGESIEDLHTCRLLIKQDIPAGLYVDPYELASLRERNITEA----------  107
RnPig-X  46  ----FL--KDGFHRDLLIKVKFGESIEDLQTCRLLIKHYIPTGLFVDPYELASLRERNITEA----------  101
ScPbn1  217  TTSLYLNEPIGLHPKIMIDL---TDFEERPKCMYLMHLQLPLELFIDKFQSSPLL-----------------  268

TbPBN1  116  LRISSDYFFDIEAPVFHVGYEVNRVNITFERLPSASRRYSNGSAAAIDIFASADDARGRLLVPIHARYEEVD  187
HsPig-X 108  --VMVSENFDIEAPNYLSKESEV---LIYAR----------RDSQCI--------DCFQAFLPVHCRYHRPH  156
RnPig-X 102  --VMVSESFNLEAPNYLSTESAV---LIYAR----------QDAQCI--------DCFQAFLPVHYRYHRPH  150
ScPbn1  269  ----LFGEDDLELPEYSLRDKAWGSESIFEL----------K-------------AGTMNEVTLHTRYIEPS  313

TbPBN1  188  STTPFSLQAFFS--------RNTSVRRCIPSITVRGVV-VDRGGRCVKFVSALMKRYDDALTSSLTSPPVSV  250
HsPig-X 157  SEDGEASIVVNNPDLLMFCDQEFPILKCWAHSEVAAPCALENEDICQWNK----MKYKSVYKNV--------  216
RnPig-X 151  KKDGDTLIVVNNPDLLMHCDQEFPILKCWAQSEVAAPCSLKSEEICQWKN----MQYKSILKNL--------  210
ScPbn1  314  NNKGDKLEVSFDPEVILACDTGDNKVSRNP---------------FYKKG----LGYESLFTDDTTFRHL--  364

TbPBN1  251  LDGNIPETSPGCRNVPVGLLSSLRVVYT--TVVALQCIGAAIVILSLLL--L------------  298
HsPig-X 217  ------------ILQVPVGLTVHTSLVCSVTLLITILCSTLI--LVAVF--KYGHFSL-------  258
RnPig-X 211  ------------TVQVPVGLTIHTSLVCSVTLLITVLCSTLI--LLAVF--KYGHFSL-------  252
ScPbn1  365  ------NSTTLLVPIPRPDTKDYSKIKNG----TLLCLLIS--IIYIFSKVFGNNKKKRSVKRE  416
```

Figure S3: Alignment of the amino acid sequences of TbPBN1 and the human (Hs), rat (Rn) and *S. cerevisiae* (Sc) homologues. Conserved residues are indicated by purple shading, the predicted C-terminal transmembrane domains are underlined in black, and the N-terminal signal peptides of TbPBN1, and human and rat Pig-X, are underlined in red.

Table S1: List of primers used in this study

No.	Primer Name	Primer Sequence
1	GPI14 KO Fwd	GATAGAGGCTTATCAGCTAGAAAATTTCCGGTATAATGCAGACCTGCTGC
2	G14 KO Rev	ACCGCGAAGCAAACACCCACTTGCAAGCCACCGGAACCACTACCAGAACC
3	GPI14 5' sgRNA	GAAATTAATACGACTCACTATAGGAGACGTGGTGGAAAACTACAGTTTTAGA GCTAGAAATAGC
4	GPI14 3' sgRNA	GAAATTAATACGACTCACTATAGGTGTTGTCGATGTCTTACAGTGTTTTAGA GCTAGAAATAGC
5	G00	AAAAGCACCGACTCGGTGCCACTTTTTCAAGTTGATAACGGACTAGCCTTAT TTTAACTTGCTATTTCTAGCTCTAAAAC
6	GPI14 nTag Fwd	GATAGAGGCTTATCAGCTAGAAAATTTCCGGTATAATGCAGACCTGCTGC
7	GPI14 nTag Rev	CACCGTGTCTATAAGCGACTGCAGTTCCATACTACCCGATCCTGATCCAG
8	PBN1 cTag Fwd	GCTATTGTTATTTTGTCGCTGTTATTGTTGGGGCCCCCCCTCG
9	PBN1 cTag Rev	TATGCACGAATGTAAACCAACCGACAGCCCGGCCGCTCTAGTGG
10	PBN1 3' sgRNA	GAAATTAATACGACTCACTATAGGGCAAACAGATAATTGAATACGTTTTAGA GCTAGAAATAGC
11	PBN1 KO Fwd	AAGAATTGGGACCACGCAGTTTTAGCTCCTGTATAATGCAGACCTGCTGC
12	PBN1 KO Rev	TATGCACGAATGTAAACCAACCGACAGCCCCCGGAACCACTACCAGAACC
13	PBN1 5' sgRNA	GAAATTAATACGACTCACTATAGGGGAACAATGCGGAACAAAAAGTTTTAGA GCTAGAAATAGC
14	PBN1 sense Fwd	ACAGTGACAAGCTTTGCAGTTGGAGGTCGAG
15	PBN1 sense Rev	GTGCATAGTCTAGAGCGGCTAAAGAATGCCTG
16	PBN1 antiS Fwd	GTATCGATCTCGAGGCGGCTAAAGAATGCCTG
17	PBN1 antiS Rev	TGTATCGTGGATCCTGCAGTTGGAGGTCGAG
18	GPI14 5' UTR	CCTTCCACCTTGTCATTC
19	GPI14 3'UTR	TAATACGGTCGACGCTTTC
20	GPI14 ORF Rev	GTTGAGGTACTGTTGTC
21	Hyg Rev	GATGTTGGCGACCTCGTATT
22	G418 Rev	GCTCTTCGTCCAGATCATCC
23	TbGPI14 Ect Fwd	GACTAGCACATATGATGGAACTGCAGTCGC
24	TbGPI14 Ect Rev	GTCGATTCGGATCCCTAAGCTACTTTGATCGAC
25	ScGpi14 Ect Fwd	GACTAGCACATATGATGACTGGCGAAGAATGG
26	ScGpi14 Ect Rev	GTCGATTCGGATCCTCAGTTGTTCTTTTTGTTG
